# Viral diversity in children with diarrhea in Gambia

**DOI:** 10.1186/gb-2011-12-s1-p2

**Published:** 2011-09-19

**Authors:** Irina Astrovskaya, Bo Liu, Mihai Pop

**Affiliations:** 1 University of Maryland Institute for Advanced Computer Studies, University of Maryland, College Park, MD 20910, USA

## Background

Despite a decrease in the rate of mortality due to diarrhea in the past few decades, diarrhea remains one of the leading causes of childhood deaths worldwide, especially in developing countries. The known causes of disease include infection with bacteria (for example, *Salmonella* or *Shigella*), viruses (for example, rotaviruses, noroviruses or hepatitis viruses) or parasites (for example, *Giardia lamblia* or *Cryptosporidium*); however, the true agent remains unknown in up to 40% of clinical cases [1].

Recent advances in sequencing technologies allow us to explore microbial diversity in a sample, making metagenomic analysis a promising technique to characterize the viral spectrum (that is, the viral sequences and their abundances) in stool samples. By studying the genomes of particular viruses that are present *in vivo*, we may obtain a complete picture of the causes of diarrhea and potentially identify unknown viral pathogens.

## Methods

In this project, we explored viral communities present in diarrheal samples from 40 Gambian children of 18 months of age or younger. Each sample contained 4,829 to 57,778,454 pyrosequencing shotgun reads with read lengths varying from 50 to 930 bp.

In our pipeline, we first assembled the genomes of known diarrhea-causing viruses by aligning the reads with the available references in the National Center for Biotechnology Information database and reconstructing the haplotypes from the mapped reads. Additional care needs to be taken for RNA viruses because they exist as a set of closely related but nonidentical genomes (quasispecies). We therefore reconstructed the set of the most plausible haplotypes [2] rather than the consensus genome. Next, we estimated the abundances of the assemblies by employing an expectation-maximization algorithm that takes into account sequencing error, as well as mark reads that are not adequately covered by the assemblies. Then, we focused on assembling the uncovered reads and identifying them. Finally, we analyzed the viral spectrum across all of the samples to decide whether specific genomes are responsible for causing diarrhea.

## Results

We were able to detect and assemble sequences from known diarrhea-causing viruses (such as rotaviruses, adenoviruses and noroviruses), known human viruses (such as herpesviruses and enteroviruses) and potential diarrhea-causing viruses (such as bocaviruses, astroviruses and parechoviruses). These findings were consistent with independent virology results.

In some clinical cases, sequences from classic viruses were found, but the virology results were negative.

## Conclusions

Annually, diarrhea causes about 1.8 million deaths worldwide. Although many causative agents are known, as many as 40% of clinical cases are attributed to unknown viral pathogens. The metagenomic analysis of pyrosequencing data allows us to investigate the role of viruses in causing diarrhea.
